# Experiences of nurses caring for mental health care users in an acute admission unit at a psychiatric hospital in the Western Cape Province

**DOI:** 10.4102/curationis.v38i2.1509

**Published:** 2015-12-18

**Authors:** Zintle C. Sobekwa, Sathasivan Arunachallam

**Affiliations:** 1 Lentegeur Psychiatric Hospital, Cape Town, South Africa; 2 School of Nursing, University of the Western Cape, South Africa

## Abstract

**Background:**

Caring for mental health care users (MHCUs) with mental illnesses is a major task that confronts nurses globally. It has been argued that caring for this group of patients is accompanied by unique challenges. Despite the available abundance of data about nursing patients suffering from mental illnesses, little is known about the lived experiences of nurses who care for MHCUs in acute admission units in the Western Cape province.

**Objectives:**

This study’s aim is to explore and describe the lived experiences of nurses who care for MHCUs in an acute admission unit at a psychiatric hospital in the Western Cape province.

**Methods:**

A qualitative, descriptive, phenomenological study was conducted. A purposive sampling procedure was applied which resulted in a sample that comprised eight nurses. In-depth, individual, semi-structured interviews were conducted with these eight participants. Interviews were audio-recorded and transcribed verbatim and the researcher utilised Collaizzi’s method to analyse collected data.

**Results:**

Both positive and negative experiences were reported. Positive experiences were the recovery of patients, teamwork, and passion for caring. Negative experiences were the feelings of being unappreciated and unsupported by authorities. Physical assault by MHCUs, shortage of staff, increased workload and burnout was also reported.

**Conclusions:**

In-service training about management of aggression needs to be provided, debriefing sessions to deal with burnout needs to be arranged, and research to quantify levels of burnout should be conducted.

## Introduction

Internationally, nurses’ experiences in different clinical areas of practice have received increasing attention in recent years. Evidence from developed countries shows interesting results about the experiences of nurses in acute admission units (Zarea et al. 2012:2). However, in developing countries, such as South Africa, little is known about the experiences of nurses who care for acutely ill psychiatric patients in acute admission units (Ngako, Van Rensburg & Mataboge 2012:1). Most studies that have been conducted amongst nurses in South Africa focused mainly on experiences of nurses in general health care settings. Less studies have been conducted to explore and describe the experiences of nurses in psychiatric settings, particularly in acute admission units.

South African nurses are at the forefront of the health care system in the country, including the mental health system (South Africa, Department of Health [DOH] 2008:8). Nurses provide daily psychiatric care to people with psychiatric problems in communities and in inpatient units at psychiatric hospitals (Western Cape Department of Health 2009:11). They work in challenging and uncompromising acute inpatient psychiatric units which demands different levels of specialised care (McKinley 2009:1; Mullen 2009:83). Janse Van Rensburg (2010:383) shares this view and adds that nurses who are working in acute psychiatric units are not only faced with the challenge of having to provide care to the acutely ill patients, but there is also a severe shortage of nursing personnel in acute psychiatric units. These units are extremely busy, high pressured environments and nurses working in these units have to deal with patients who suffer from complex mental health problems. Studies carried out have shown that there is a close relationship between high workload and low job satisfaction amongst nurses at psychiatric hospitals (Konstantinos & Ouzouni 2008:188; Mohadien 2008:52).

Allen and Jones (2002:458) and Mavrogiorgou, Brüne and Juckel (2011:222), agree that acute mental health care services are in crisis because staffing and the severity of patients’ conditions require immediate attention. The authors support the fact that the acute psychiatric environment presents a major intrinsic stressor to patients who are experiencing delusions, hallucinations, and other psychiatric problems. They add that these conditions have a severe impact on service delivery and patients’ outcomes.

### Problem statement

In the field of mental health practice, it is known that caring for acutely ill MHCUs is a difficult task confronting clinicians, particularly nurses (Mullen 2008:83). Nurses who care for patients in acute psychiatric units work in an extremely busy and high pressured environment and are faced with a challenge of having to deal with patients who suffer from complex mental health and psychiatric problems (McKinley 2008:1). Despite the long hours spent by nurses who care for acutely ill MHCUs in acute admission units, little is known about the lived experiences of these nurses who are working at a psychiatric hospital in the Western Cape province.

## Aim of the study

The aim of the study was to explore the lived experiences of nurses who care for mental health care users (MHCUs) in an acute admission unit at a psychiatric hospital in the Western Cape province.

### Objective of the study

The objective of this study is to describe the lived experiences and feelings of nurses who who care for MHCUs in an acute admission unit at a psychiatric hospital in the Western Cape province.

### Definition of key concepts

#### Experiences

An event or occurrence which leaves an impression on someone or others (Oxford Learner’s Dictionary 2014, para. 2). In this article, the term will refer to events or circumstances experienced by nurses whilst caring for acutely ill psychiatric patients.

#### Caring

A process of providing physical and psychological care to patients with mental disorders (The Free Dictionary 2002, para. 2).

#### Nurse

A person registered under Section 31 (1) of the *Nursing Act* No 33 of 2005 and who practises nursing or midwifery (South Africa 2005:6). In this article, a nurse refers to a professional nurse and an enrolled nurse who provides care to acutely ill psychiatric patients.

#### Mental health care user

A person who either receives care, treatment, and rehabilitation services, or who uses a health service at a health establishment that aims at enhancing the mental health status of a user (South Africa, *Mental Health Care Act* no. 17 2002:10). For the purpose of this study, the term MHCU(s) is used interchangeably with patient(s).

#### Acute admission unit

An acute psychiatric unit in a psychiatric hospital where acutely ill patients are admitted in order to receive short mental health care, treatment and rehabilitation (Moosa, Jeenah & Raghubir 2004:27). In this article, admission unit refers to both male and female acute admission units.

#### Psychiatric hospital

A health establishment that provides specialist mental health care, treatment, and rehabilitation services to people who require such services (Cullinan 2006:18).

## Literature review

### Prevalence of mental disorders

According to the World Health Organisation (WHO) (2011:13), neuro-psychiatric disorders are estimated to contribute to 13% of the global burden of disease and the figures are expected to increase by 15% in 2030. It is estimated that 450 million people worldwide have a mental disorder (WHO 2009:3). According to Bradshaw (in Corrigall et al. 2007:4), neuro-psychiatric disorders account for the second highest proportion of the local burden of disease in South Africa, after HIV/AIDS. A large scale study conducted by Herman et al. (2009:340), found that the life-time prevalence of mental disorders in South Africa is 30.3% with the Western Cape having a life-time prevalence of 42%; the lowest prevalence occurs in the Northern Cape at 29%. The study also finds that the most prevalent life-time mental disorders in South Africa are anxiety disorders (15.8%) and substance use disorders (13.3%). It is estimated that mental disorders in the Western Cape are rated second in the top five major contributors to the burden of diseases (Corrigall et al. 2007:4). The most prevalent life-time mental disorder in the province is substance abuse with a rate of 20.6% (Herman et al. 2009:340). The significant increase in the prevalence of mental disorders has exerted extreme pressure on acute psychiatric services in all provinces of South Africa, especially in the Western Cape (Janse Van Rensburg & Jassat 2011:22–23).

### Acute psychiatric units

Patients are usually admitted to an acute psychiatric unit when they are significantly disturbed, vulnerable and mentally ill according to Deacon, Warne and McAndrew (2006:752). These patients present a variety of complex psychiatric problems that often requires skilled and competent clinicians. Mullen (2008:85) emphasises that acute inpatient units are challenging environments to work in. According to Emsley and Pienaar (2005:133), the most common psychiatric problems and emergencies that patients often present in acute psychiatric units are aggression and restlessness, suicidal ideation, drug induced psychotic episodes, acute grief reaction and crisis management. Mullen adds that these units are extremely busy, high pressured environments that provide care to acutely ill patients and can be challenging. Nurses always have the first contact with these patients and subsequent care is also provided by the nurses. Nurses who work in these units deal with several other challenges that include high bed occupancy rates, increased patient turnover, and short lengths of stay (Mullen 2008:83).

## Research design and method

A qualitative research approach and descriptive phenomenological research design was used in order to explore and describe the experiences of nurses caring for MHCUs in an acute admission unit at a psychiatric hospital.

### Population and sampling

This study comprised all nurses working permanently with MHCUs in two acute admission units of a psychiatric hospital in the Western Cape. A purposive sample was used to select participants from the two acute admission units. A purposive sample of 8 nurses was drawn from the population of 24 nurses.

### Data collection method

This study used in-depth, semi-structured, one-on-one interviews to explore and describe the experiences of nurses caring for MHCUs in an acute admission unit. The researcher’s focus was on the experiences of nurses who care for MHCUs; this method allowed the researcher to expand beyond the main question through probing. De Vos et al. (2011:353) assert that semi-structured interviews are useful when the researcher intends to gain a detailed picture of the participants’ beliefs, perceptions, and accounts with regard to a particular phenomenon. Connelly (2010:127) states that in phenomenological studies, interviews are a widely used method of data collection. An interview schedule was used as a guide for the progress of the interviews. The interview consisted of open-ended questions that where followed by probing questions in areas where further clarity was needed. Interviews were conducted at the hospital in an office. This approach is in line with the nature of phenomenological research where the researcher studies participants in the environment where the phenomenon of interest takes place. All interviews were audio-recorded and transcribed verbatim. Field notes were also collected.

### Data analysis

Collected data from individual one-on-one in-depth interviews were analysed by using Collaizzi’s (1978:48) seven steps method of data analysis. Data analysis was performed after data collection. Analysis involved reading all participants’ description of the phenomenon. The researcher then returned to the original transcripts and extracted significant statements, spelt out the meaning of each significant statement, organised the aggregate formalised meanings into clusters of themes (coding), wrote an exhaustive description, and consulted all the participants to validate of the descriptions (member checking). The researcher made use of the services of an independent coder to verify the codes generated by the researcher.

### Purpose of the study

The purpose of the article is to explore and describe the experiences of nurses who care for MHCUs in an acute admission unit at a psychiatric hospital.

## Ethics considerations

Ethics clearance to conduct the study was granted by the Senate Research Committee of the University of the Western Cape. Ethics approval was also granted by the Ethics Committee of the hospital concerned. Participants were fully informed about the nature, purpose, objectives, benefits and risks of the study. Participants were informed that participation in the study was voluntary and that they could withdraw from the study at any time. A consent form was signed by all participants. Should any participant become emotionally affected, then the services of ICAS (Employee Well-being Programme) will be utilised. This service is for Western Cape government employees and includes psychologists and social workers. The researcher also ensured the audio-recordings, field notes, and transcripts were stored safely in a locked cabinet and could only be accessed by the researcher. Pseudonyms were used to protect participant’s identities:

- **Trustworthiness** was assured by adhering to the requirements of an empirical enquiry (Burns & Grove 2005:75). Bracketing contributes to trustworthiness by helping the researcher ensure that their beliefs do not influence the collection of the data and it analysis.
- **Confirmability** is concerned with the degree to which the results could be confirmed by other people. To ensure that the study results were confirmable, the researcher documented the procedures, as well as checked and rechecked the data throughout.
- **Transferability** was ensured by providing a description of the research setting, study participants, and a thick description of data.
- **Credibility** **was** achieved by multiple reviews of the audio-recordings, field notes, member checking, and the presence of the independent coder during data analysis.
- **Dependability** was achieved by the research plan being carefully documented, triangulation methods used, and the researcher’s role described.

## Discussion of results

After the completion of data analysis, five themes emerged from the interviews which constitute the findings of the study. These are highlighted in Table 1.

**TABLE 1:** Summary of themes that emerged during the study.

Themes	Sub-themes
Positive job aspects	recovery of MHCU’s team work (intergroup relations passion for caring
Unsafe working environment	fear of assault by MHCUs aggressive MHCUs unpredictable MHCUs
Challenging working environment	increased patient numbers shortage of staff overworked & burnout
Compromised clinical care	compromised nursing care
Challenging experiences	lack of support from authorities feeling unappreciated by management

### Positive job aspects

Despite some negative experiences reported by participants in the study whilst caring for MHCUs in an acute admission unit, there were three key positive sub-themes that nurses reported about caring for MHCUs.

### Recovery of MHCUs

Nurses who participated in the study reported that MHCUs’ recovery was an aspect that they viewed as being positive about their work. One participant said that it was really rewarding for her to see patients recovering:

‘Positives yoh there’s a lot of positive things … um … positive things … mostly when our patients are getting feeling better. It’s rewarding … Um.’ (P2)

Most participants felt that recovery of MHCUs was a reward for their work despite the challenging circumstances they worked under:

‘In a nice capacity you see the value of work … but on the other side, what makes me feel good is when you can see the improvement … if there’s an improvement and really in ward five you can see.’ (P1)

Challenges included shortage of staff, workload, and feelings of not being supported by the authorities. One participant said that MHCUs’ recovery was an outcome of the continued teamwork in the acute unit. That finding was consistent with the findings of Mohadien (2008:63) who investigated factors that influenced job satisfaction amongst nurses at the same psychiatric hospital where this study was conducted. Her study found that nurses felt relieved by the positive outcomes of patient care. Furthermore a study conducted in Ghana by Jack et al. (2013:5) states that mental health workers at three psychiatric hospitals described positive patient interactions of feeling fulfilled when their patients recovered. The signs of recovery of the patients included leaving the hospital, returning to their family, getting a job and dressing appropriately.

### Teamwork

It was found that nurses in the acute admission unit where the study was conducted reported the significance of working as a team and assisting one another in overcoming the difficulties that each day presented:

‘[*T*]eamwork is nice and that makes everything positive and you feel that you can work with all your patients and when-I think when-when-when you-when you and your team are-are positive you project that feelings on your patients so you will have more calm patients.’ (P2)

The multiple functions and benefits of teamwork in an acute mental health ward are considered vital and are recognised by mental health nurses (Cleary et al. 2011:457). Effective team work and good relationships with colleagues were the most highly valued positive influences on morale as stated by Totman et al. (2011:4).

One participant said:

‘We work as team; we are like a family here.’ (P8)

The participant continued and said that nurses stood together at all times. One participant gave an example of the situation where an MHCU would become aggressive and, therefore, required seclusion. The participant said that the nurses came together in an effort to calm the patient down and assist her with getting into a seclusion room. This finding was consistent with that of Shattel, Andes and Thomas (2008:247) who conducted a study about how patients and nurses experienced the acute psychiatric environment in an overseas hospital. They found that despite working under compromised conditions, team work amongst nurses gave them mental strength. In line with the findings of this study, they also found that nurses supported one another with patient care and assisted one another in situations that demanded teamwork in supporting MHCUs. In this study, participants reported that they received emotional support from the team during difficult times. For example, one participant stated that after suffering an assault by an MHCU, she received intensive support from her nursing colleagues to cope with the experience. It appeared that the strong culture of teamwork amongst the nurses in the acute unit enhanced a spirit of unity amongst the nurses:

‘We are working as a team! We are working as a team.’ (P8)

One participant attributed the recovery of MHCUs to the strong teamwork that was a norm in the unit. This finding was consistent with the findings of Kalisch, Lee and Rochman (2010:944) who conducted a study to explore the influence of the unit characteristics, staff satisfaction, and job satisfaction of nurses in their current positions and occupations in an acute care unit. Their study found that there was a higher level of team work reported by nurses that enhanced nurses’ job satisfaction, particularly in cases where staffing was adequate. However, in this study nurses reported high levels of staff shortages that did not seem to influence teamwork amongst nurses in the unit. The findings of this study concur with the findings of Mohadien (2008:112) that reveal that good interpersonal relationships amongst nurses enable nurses to work together in a team context.

### Passion for caring

Nurses in this study reported that despite working in a challenging environment with negative experiences at times, they remained passionate about the work that they were doing:

‘I love it! I love it, I love it! Um … how can I say … dealing with acutely ill psychotic patients because … I love psychiatry and I love admissions … I enjoy it but you take time … hey getting into you … [*Laughs*].’ (P2)

Some participants reported that although they had been assaulted by MHCUs and somehow felt unappreciated and unsupported by authorities, they enjoyed caring for MHCUs and viewed their job as interesting. A study in Iran by Zarea et al. (2012:128) revealed that the psychiatric nurses enjoyed caring for patients without expecting any reward or appreciation. They mentioned that they had the feeling of a sense of duty, honourable income, and the feeling of self-sacrifice which motivated them to deliver nursing care to their psychiatric patients. This shows that despite the challenges, there are nurses that have a passion for caring for psychiatric patients.

### Unsafe working environment

This theme comprised nurses’ experiences of the factors that they felt made the acute psychiatric environments unsafe to them. The aggression, unpredictability, and assaults of nurses by MHCUs were discussed and emphasised. Nurses in this study perceived the acute admission unit as unsafe and reported that this part of their experience was uncomfortable.

### Unpredictability, aggression, and assault by MHCUs

The findings of this study showed that nurses experienced aggression and assault by MHCUs who they believed were unpredictable most of the times, as stated by one participant:

‘Ah, about aggressive … sometimes you feel nervous, jha … you feel nervous because you don’t know what the patient is going to do.’ (P4)

Both male and female nurses reported that they had experienced verbal and physical assault from MCHUs who they cared for in acute admission units at the hospital. Threats, violence and assaults in the workplace are also considered a significant source of stress in psychiatric nursing. Roche and Duffield (2007:97) noted that violence and threats of violence have been identified as particularly stressful to nurses working in mental health, with workplace violence having a direct impact on them. Nurses felt unsafe at times in the ward, particularly female nurses who felt targeted by male MCHUs as stated by one participant:

‘Especially males … you get scared because you know you won’t even be able to handle this person, the only thing that you can do is speak to him but if even from there he does not listen … you need to know that you must keep a distance because he can do anything. They are unpredictable, you don’t know what he will do now and what he won’t do now … so you must always stay wide awake all the time but hey … we must be here for them.’ (P3)

Nurses in this study perceived aggression as offensive, humiliating and unacceptable but understood that it was a result of MHCUs’ psychiatric illness. One participant relayed an incident when a female nursing staff member had been physically assaulted with a chair by a MCHU. He stated that female nurses were sometimes helpless in the unit and needed protection by the male nurses. This finding was consistent with the findings of the study conducted at a public mental health hospital in Pretoria by Ngako et al. (2012:7). Their study investigated the experiences of psychiatric nurse practitioners who were working with MHCUs with acute symptoms. They found that nurses experienced a sense of helplessness, fear and feelings of frustration (Ngako et al. 2012:9). These findings exist in many other studies. One such study was conducted in Nigeria by James, Isa and Oud (2011:131) to investigate how a sample of nurses experienced aggression at two psychiatric facilities. Consistent with this study, their (James et al. 2011:132) study found that verbal aggression was one of the most common types of aggression experienced by nurses. In their study they also reported that male nurses experienced physical violence and other aggressive behaviour.

One of the participants in this study provided an example of a male nurse who was bitten by a MCHU who had a contagious disease; this type of physical violence was emphasised by James et al. (2011:132). Another study by Duxbury (1999:110) concurs with the findings of this study by explaining that verbal abuse and physical abuse were commonly encountered by registered nurses whilst caring for MCHUs in acute psychiatric unit. One aspect discussed by James et al. (2011:132) in their study was that of sexual intimidation. In contrast with their finding that sexual intimidation was common, none of the participants in this study experienced sexual intimidation by MHCUs. Another recent study conducted by Ngako et al. (2012:5) in Pretoria that investigates the experiences of psychiatric nurse practitioners working with MHCUs who present acute symptoms also finds that nurses experience working with acutely ill MCHUs as unsafe. Their study also emphasises that sexual harassment is commonly reported by female nurses, particularly during weekends. As pointed out before, female nurses in this study did not report such experiences.

### Challenging working environment

This theme described challenges that nurses face on a daily basis whilst providing psychiatric and mental health care to acutely ill MHCUs in an acute admission unit. Those challenges had an impact on the nurses and, therefore, shaped some of their experiences. Those experiences were frequent and nurses reported that they had an impact on the delivery of quality nursing care.

### High patient turnover and shortage of staff

This study found that one of the challenges faced by nurses in the acute admission unit was the shortage of staff. Nurses felt that despite having to care for almost 30 challenging MHCUs in the unit, the staffing was not adequate to properly care for the MCHUs in the unit:

‘Draining because why … most of the times its short-staff with the capacity of 30–35 patients, um. Only one professional nurse on duty, sending in lot of students without experienced nurses … so then you get burned out!’ (P2)

Nurses reported that inadequate staffing of the unit was a major challenge, as that made it difficult for them to attend to all MCHUs. This finding was consistent with those of Totman et al. (2011:3) who investigated the factors that had affected morale of the staff in inpatient wards in England and found that nurses had reported a severe shortage of nursing staff which had a negative impact on the staff morale. Furthermore, nurses in this study reported that it was difficult at times for them to care for large numbers of MCHUs whom they had to admit. Some nurses who participated in the study reported that the issue of a staff shortage was a great concern to them and they believed that it had a negative impact on MCHUs outcomes. According to the Department of Health (2008:11), the shortage of nurses in South Africa is a serious challenge that the health care system faces, as it has a negative effect on the delivery of quality health care. Moreover, the Western Cape Department of Health (2009:10) in their *Provincial Nursing Strategy* also reported that the shortage of nurses, particularly trained psychiatric nurses was the issue that affected delivery of quality mental health care the most. Nurses complained that nurse-patient ratios were completely abnormal and the delivery of quality nursing care was ‘impossible’, as expressed by one participant:

‘Like for nursing care to be rendered or to be promoted, you have to … you’ve got to keep the nurse-patient ratio rate … and that here in female admissions unit is very … We very few … like I said most of the times like I said we deal with shortage of staff.’ (P2)

This finding concurred with those of Mohadien (2008:54) who found that there was a shortage of nursing staff in the entire hospital, and argued that optimal MCHUs care would be affected by such shortage. This shortage of nursing staff contributed to an increased workload that, in turn, contributed to low staff morale. Shortage of staff, high numbers of MCHUs in the unit, and feelings of being not appreciated and supported, all seem to have contributed to increased levels of burnout. This finding was consistent with those of Currid (2009:44) who found that poor staffing levels, huge workloads, and a high number of MCHUs that exceeded standard limits, contributed to the nurses’ feelings of being under pressure.

### Overworked and burnout

The study found that nurses who participated in the study experienced being overworked. Some participants reported that the workload was unbearable, as they had to work with high number of MCHUs as a result of high daily admission rates:

‘Like … Like you get tired and burned out … and you short of staff and you tend to get irritable and then you shift that feeling to your patients and its havoc.’ (P2)

They also reported the experience of working with challenging MCHUs in the context of extreme staff shortage that exerted too much strain on the nurses. This finding concurred with the study conducted by Sherring and Knight (2009:1239) that explored burnout amongst mental health nurses in London, and found that 41.0% of mental health nurses in the city experienced high burnout and emotional exhaustion scores. In addition to the prevailing findings, one participant stated that she felt:

‘… drained, got irritated at times, and projected such feelings to [*sic*] patients which created havoc.’ (P2)

Few other studies nationally and internationally have reported that an increased workload contributes to exhaustion and burnout amongst nurses in psychiatric and general units. An example is the study by Mohadien (2008:64) that finds that 57.38% of nurses, particularly enrolled nurses and enrolled nursing assistants, report levels of being overworked and feelings of tiredness. One of this study’s participants stated:

‘Tired! You get tired because you got have to do everything, you got to do your administration work, you got to do your supervision work, you have to give the patient quality care and with the … nurse-patient ratio here is very bad. So you get yourselves to do things that … you … that are impossible, giving attention to 35 patients at once. And acutely psychotic patients!’ (P2)

Currid (2008:40) found that workload and lack of staff levels in their acute units was inadequate, workloads were overwhelming and constant which resulted in stress.

This finding was consistent with the finding of Mohadien (2008) who also found in the same setting that nurses experienced increased levels of burnout and exhaustion whilst caring for MHCUs. However, it should be noted that this study only focused on the experiences of the nurses who were caring for MHCUs in an acute admission unit, whereas Mohadien’s study focused on job satisfaction of a sample of nurses. One participant in the study reported that, at times, they had to work overtime when they were supposed to be off-duty to assist their colleagues who he believed would have been exposed to risk should they not come to work:

‘I’ll make you an example, like now … here in the ward we have 34 patients and that thing has been happening since the beginning of this year. The capacity of this ward its 30 … but it has been happening … yes we understand that they sometimes they call overtime but now when they call overtime, when do those people rest? Then the burnout syndrome starts now!’ (P7)

He further asked when they would rest if they had to come to work even during the time that they had to be off. He stated that such a situation was the beginning of burnout amongst staff. This finding concurred with the findings of Jenkins and Elliott (2004:622) who conducted a study aimed at investigating and comparing levels of stressors and burnout of qualified and unqualified nursing staff in acute mental health settings. Their study found that qualified nurses reported higher levels of workload stress than unqualified nurses. Furthermore, their study also revealed that almost half of the nursing staff showed high burnout in terms of emotional exhaustion.

### Compromised clinical care

Participants reported that quality MCHUs care was hindered by challenges such as a high workload and burnout and felt that positive MCHUs outcomes were at times not achieved. This theme emerged as one of the frequent and important themes in this study, as it related to nurses’ core business in the unit.

### Compromised nursing care

Participants also reported concerns about the quality of nursing care provided to MHCUs in the admissions unit. Participants, particularly professional nurses, expressed concerns about what they described as ‘poor quality nursing care’:

‘For example if maybe I am interviewing a patient now, there is my other patient that side is doing something, I have to run and fetch his nursing process and attend to him and write whatever he did or else if I don’t write it I will forget it and now his treatment plan is going to be affected because there is data that I have not collected …’ (P8)

They believed that staff shortages, high MCHUs turnover, and increased work load were factors that contributed to poor nursing care. This finding concurred with the findings of Ngako et al. (2012:7) who found that nurses had reported that nursing care provided to MHCUs was threatened by challenges that confronted the nurses in the acute psychiatric unit.

One participant complained about a lopsided nurse-patient ratio during the interviews. She claimed that at times, they would be only three nurses on duty to care for 30 MHCUs:

‘Like I said we deal with shortage of staff, then there’s escorting, so you having experienced nursing staff going with your psychotic patients, acute psychotic patients.’ (P6)

Other participants reported that providing quality nursing care under such circumstances was difficult, but they tried their best. This finding was consistent with those of Currid (2009) who conducted a phenomenological study about the experiences of stress amongst nurses in acute mental health care settings in London. Currid found that nurses reported that lack of resources, such as staff shortages in acute units, had a negative impact on MCHUs’ care. Cleary (2004) also found that nurse-MCHUs interaction in acute units was affected as a result of increased workload.

Continuing with describing their experiences in relation to nursing care provided, one participant stated that, because of pressure and changes in hospital policies, they were at times forced to transfer some MCHUs to other wards prematurely. The participant claimed that some were still acutely ill or psychotic and still required stabilisation. However, the early transfers happened as a result of the admission of other psychiatric cases, such as emergencies, failed discharges, and recurrent readmissions. One of the participants attributed the issue of readmissions to the frequent admissions of MHCUs who suffered from substance induced psychosis, an occurrence that was escalating in the catchment area of the hospital. That meant that, in addition to shortage of staff in the units, the workload of nurses increased as the number of readmissions increased. This finding was consistent with Mohadien (2008:113) who reported that nurses were dissatisfied with increased MCHUs numbers and felt unable to provide adequate care for them.

### Challenging experiences

The participants reported challenging experiences, such as a lack of support from authorities and feelings of being unappreciated.

### Lack of support from authorities and feelings of being unappreciated

Nurses who participated in this study reported a lack of support from the management of the hospital and of feeling unappreciated:

‘No … I was still saying … the managers when something happens to the patient they are very quick to come and ask where were the nurses? What where they doing? …. but when the nursing staff gets assaulted not even a single person comes to enquire how is that employee doing today, and perhaps make a follow up the next week on the injured employee or even just doing what is called ‘debriefing’ for all the staff members.’ (P3)

This finding was consistent with the findings of Sherring and Knight (2009:1239) who found that nurses who felt unsupported and undervalued became demotivated and suffered from burnout. Nurses expressed the fact that they felt unappreciated for their efforts despite working under compromising circumstances with shortage of staff, increased work load, and demanding MCHUs. Participants in the study believed that the hospital authorities somehow neglected them under difficult circumstances.

This finding was consistent with the study of Mohadien (2008:60) who found that 50% of nurses at the same hospital felt unappreciated and reported that they were not recognised for their extra efforts under compromised working conditions. To illustrate that there was a lack of support from hospital authorities, one participant stated that there were no debriefing sessions available to assist staff members who, for example, experienced trauma following an assault by a MCHUs. However, it is worth mentioning that participants recognised and acknowledged the support services offered by the Work Capability Assessment (WCA) in such cases, but felt that the intervention was not enough, as it focused more on physical injuries and issues of compensation. One participant reported that ‘emotional injuries’ experienced by staff members who suffered an assault were not well attended to. This finding was consistent with the findings of Ngako et al. (2012:7), as they also found that psychiatric nurse practitioners who participated in their study expressed a need for emotional support from the authorities. Participants pleaded for more emotional and psychological support. That lack of support and feelings of not being appreciated were a dominating theme:

‘We do not want ICAS, we want the managers of this hospital to come to us as people who care for us to come and speak to us to find out how we feel.’ (P7)

Nurses in acute admission units expressed negative attitudes towards the management as well as feelings of anger, blame and hopelessness. Some nurses in the study complained that the management had at times failed to attend to their requests when they needed additional nursing staff when there was a shortage of staff.

### Limitations of the study

The results of this study were limited to nurses who worked in two acute admission units at a psychiatric hospital where the research took place during the period of the study. Therefore, its findings cannot be generalised to other acute admission units of other psychiatric hospitals in the province or elsewhere. Furthermore, the findings of this study are only applicable to the population that was studied and not the entire population of nurses in other units.

## Recommendations

### Nursing practice

The study has identified that the shortage of staff in acute admission units of the hospital is a continuing phenomenon that requires immediate attention. The researcher acknowledges this shortage as a national and global issue that has a severe impact on the well-being of MCHUs. However, as this study and other research have revealed, acute admission units at psychiatric hospitals are challenging environments and measures should be taken to ensure that they are well staffed. It would be prudent to increase the number of nurses with advanced training in psychiatric and mental health nursing.

### Debriefing sessions for nursing staff

Given the fact that nurses in the study experienced the acute admission units as demanding and challenging with an increased workload which led to some of the nurses reporting signs of burnout and emotional exhaustion, it has become evident that there is a great need to introduce ‘debriefing sessions’ for nurses. This can be carried out as part of the formal programme of the acute units whereby formal debriefing sessions are held by specifically appointed therapists. This is necessary as the nurses work in such demanding environments with a difficult MHCU population. Nurses in the study reported experiences of being assaulted by MHCUs, and emotional injuries that manifested after assaults. Such debriefing sessions may assist the nurses to discuss their feelings with a professional counsellor who would be able to identify sources of stress and provide remedial actions. That would make the nurses feel that they are listened to and appreciated more.

### Nursing education

Like other national and international studies, this study reveals that aggression is very prevalent in acute admission units at psychiatric hospitals. It is recommended that continuous in-service training about the management of aggression is introduced and intensified at the hospital. All staff should be encouraged to attend such training. The in-service training may be carried out in terms of regular workshops by members of the multi-disciplinary team and should perhaps focus on practical aspects.

### Further research

The researcher recommends that further research be conducted on the prevalence of MCHUs’ aggression in the acute admission units at the hospital. This will allow the hospital managers to quantify the problem and determine the steps of dealing with aggression.

It is recommended that further research be conducted on the prevalence of burnout amongst nurses who work in acute admissions units at the hospital. Such research will determine whether there is indeed a need to introduce measures to deal with burnout.

Lastly, continual research is recommended about the experiences of nurses who are caring for MHCUs in other units, such as forensic psychiatry, intellectual disability, and child and adolescent units in order to have a comprehensive picture of how nurses experience their caring for MHCUs at the entire hospital.

## Conclusion

Nurses who cared for MHCUs in acute admission units of a psychiatric hospital described lived experiences. The study reported both positive and negative experiences in relation to caring for MHCUs. Nurses described the caring of MHCUs in the unit as challenging. They felt unsafe when carrying out their duties as some experienced assault by MHCUs, felt uncared for, and unsupported by the management of the institution. Nurses experienced shortages of staff whilst having to deal with increased MCHU numbers. Therefore, an increased workload remained a challenge that eventually could lead to feelings of burnout and emotional exhaustion. However, despite such negative experiences, nurses remained passionate about caring for MHCUs, and the strong teamwork that surfaced during interviews seemed to be their source of strength. The positive experiences included witnessing the recovery of MHCUs and experiencing teamwork amongst staff members.
